# Report of a Case Terminating Fatally from the Local Application of Corrosive Sublimate in the Cure of a Species of Tetter

**Published:** 1831

**Authors:** Allen Kimball

**Affiliations:** Appling, Columbia Co., Georgia


**Art. IV.**—*Report of a Case terminating fatally from the local application of the Corrosive Sublimate in the cure of a species of Tetter, (Herpes exedens.)* By Dr. Allen Kimball, of Appling, Columbia Co., Georgia.

Not long since, I was called at a late hour of the night to see Miss ———, aged about 12 years. When I arrived, I received from the mother, a brief narrative to this effect. That her daughter had been affected for a considerable time with Tetter; being over solicitous to have it cured, that she might be at school, she applied to a physician, who promised a ready cure by the corros. sublimate. The ointment was prepared, and she obtained it early that morning. She had previously received verbal directions to apply a portion the size of a large pea. By parting the hair in different places, the whole scalp was found to be almost entirely covered with a lamous eruption; and she supposed from the loose manner in which the directions were given, that there was no danger in the article, and that the directions went for the application to every part of the head much affected. At 9 o'clock of that day, she parted the hair in different directions, and freely applied (by the finger) the unguent. A few minutes only had elapsed, before the child began to "cry" of the most insufferable heat and burning, which were followed, in less then half an hour, by nausea, sick stomach, and vomiting, and threatened with convulsions, from excess of pain. Laudanum was given freely, and sinapisms to the gastric region and etxremities, with scarcely temporay ease. The mother states, that she applied forthwith to the Dr., but received assurances that the effects were only temporary. (I have been told on the ground that there were no absorbents of the head.) When I saw the child, (12 o'clock at night,) she had short intervals of ease and tranquillity, frequent vomiting, particularly if any fluid was taken into the stomach. Her head was immediately shaved and washed thoroughly. with warm soap and and water; warm starch poultices were the only application that gave much relief. From nine in the morning until the same hour in the evening, she passed upwards of one hundred ounces of urine; the pulse was frequent, and small; she had no other urinary evacuation, until the fourth evening, which was very scanty, and continued so until her death. The third day from the application, blood passed off freely per anum, and increased daily; all the glands of the jaws, &c., were swelled, with a strong mercurial fetor of the breath. These symptoms became more inveterate, accompanied with great thirst, soreness of the abdomen, &c., until the 7th day, when it terminated fatally. The mother used only the index finger in the application of the ointment, except the washing of the head about fifteen hours after. I do not know the strength of the ointment; she states, that it produced a sensation to the touch, like sand. About 3 grains were rubbed on of the preparation. She (the mother) was deeply salivated, exhibiting all the usual symptoms of a general ptyalism, which disappeared under the customary course of remedies

This case presents two lamentable circumstances: that of negligence and rashness. I do not say this with the fear of contradiction, for I am warranted in the assertion, that every member of the medical profession, is not, virtually, a member of a temperate society, and it is sorely to be regretted, that too many of us are addicted to habits of intemperance.

The great Gregory says "temperance and sobriety are virtues peculiarly required in a physician. I have heard it said of some eminent physicians, that they prescribed as justly when drunk, as when sober. If there was any truth in this report, it contains a severe reflection against their abilities in their profession. It shows that they practice by rote," &c. "Drunkenness implies a defect in the memory and judgment. It implies confusion of ideas, perplexity, and unsteadiness, &c." It is due the efficacy of this medicine, to say, that several inveterate cases have been thoroughly cured in this vicinity, by the spiritous and aqueous solution, judiciously applied several times daily. It should never be forgotten, that all the drastic poisonous medicines, require to be prescribed cautiously, not only with a regard to the quantity, but to the age, strength, and constitution of the patient. Of all classes of professional men, sobriety, discretion, and caution, are more required in the practitioner of medicine. The life of his fellow creature is constantly at his disposal: mistake, or design, sends them from whence no traveller returns. It is to be regretted, that the general consent of mankind, will not recognize drunkenness and dissipation, as a forfeiture of the medical man's profession.

